# Identification of genes related to immune enhancement caused by heterologous ChAdOx1–BNT162b2 vaccines in lymphocytes at single-cell resolution with machine learning methods

**DOI:** 10.3389/fimmu.2023.1131051

**Published:** 2023-03-02

**Authors:** Jing Li, FeiMing Huang, QingLan Ma, Wei Guo, KaiYan Feng, Tao Huang, Yu-Dong Cai

**Affiliations:** ^1^ School of Computer Science, Baicheng Normal University, Baicheng, Jilin, China; ^2^ School of Life Sciences, Shanghai University, Shanghai, China; ^3^ Key Laboratory of Stem Cell Biology, Shanghai Jiao Tong University School of Medicine (SJTUSM) and Shanghai Institutes for Biological Sciences (SIBS), Chinese Academy of Sciences (CAS), Shanghai, China; ^4^ Department of Computer Science, Guangdong AIB Polytechnic College, Guangzhou, China; ^5^ CAS Key Laboratory of Computational Biology, Bio-Med Big Data Center, Shanghai Institute of Nutrition and Health, University of Chinese Academy of Sciences, Chinese Academy of Science, Shanghai, China; ^6^ CAS Key Laboratory of Tissue Microenvironment and Tumor, Shanghai Institute of Nutrition and Health, University of Chinese Academy of Sciences, Chinese Academy of Sciences, Shanghai, China

**Keywords:** ChAdOx1-BNT162b2 vaccine, immune, lymphocyte, machine learning, scRNA-seq profile

## Abstract

The widely used ChAdOx1 nCoV-19 (ChAd) vector and BNT162b2 (BNT) mRNA vaccines have been shown to induce robust immune responses. Recent studies demonstrated that the immune responses of people who received one dose of ChAdOx1 and one dose of BNT were better than those of people who received vaccines with two homologous ChAdOx1 or two BNT doses. However, how heterologous vaccines function has not been extensively investigated. In this study, single-cell RNA sequencing data from three classes of samples: volunteers vaccinated with heterologous ChAdOx1–BNT and volunteers vaccinated with homologous ChAd–ChAd and BNT–BNT vaccinations after 7 days were divided into three types of immune cells (3654 B, 8212 CD4^+^ T, and 5608 CD8^+^ T cells). To identify differences in gene expression in various cell types induced by vaccines administered through different vaccination strategies, multiple advanced feature selection methods (max-relevance and min-redundancy, Monte Carlo feature selection, least absolute shrinkage and selection operator, light gradient boosting machine, and permutation feature importance) and classification algorithms (decision tree and random forest) were integrated into a computational framework. Feature selection methods were in charge of analyzing the importance of gene features, yielding multiple gene lists. These lists were fed into incremental feature selection, incorporating decision tree and random forest, to extract essential genes, classification rules and build efficient classifiers. Highly ranked genes include *PLCG2*, whose differential expression is important to the B cell immune pathway and is positively correlated with immune cells, such as CD8^+^ T cells, and *B2M*, which is associated with thymic T cell differentiation. This study gave an important contribution to the mechanistic explanation of results showing the stronger immune response of a heterologous ChAdOx1–BNT vaccination schedule than two doses of either BNT or ChAdOx1, offering a theoretical foundation for vaccine modification.

## 1 Introduction

The coronavirus disease 2019 (COVID-19) pandemic was brought on by the emergence of a new coronavirus strain known as severe acute respiratory syndrome coronavirus 2 (SARS-CoV-2)(1). On March 11, 2020, COVID-19 was eventually classified as a pandemic by the World Health Organization (2). As of August 12, 2022, over 588 million cases and 6.4 million deaths due to COVID-19 were reported worldwide (3). Fever, sore throat, dry cough, and pneumonia symptoms are the common clinical manifestations of the disease (4). To combat COVID-19, scientists have started working on COVID-19 vaccines. The vaccines have been injected in doses totaling over 12 billion (3). To date, several types of vaccines against SARS-CoV-2, such as RNA-based, nonreplicating viral vector, and protein-based vaccines, have been developed and are in widespread use worldwide (5).

BNT162b2 (BNT) and ChAdOx1-S-nCoV-19 (ChAd) vaccines have been the most widely used authorized COVID-19 vaccines worldwide (6). BioNTech developed BNT with the assistance of the pharmaceutical company Pfizer (7). The complete spike protein is encoded by mRNA packaged in lipid nanoparticles and modified by the addition of two prolines that stabilize prefusion conformation and improve immunogenicity to one of mRNA subunits (5, 8). The components of ChAd are chimpanzee adenoviruses (Ads) encoding the SARS-CoV-2 spike-in glycoprotein (5). Ads are double-stranded and envelope-free DNA viruses that can target a wide range of host tissues for cellular infection (9, 10).

BNT and ChAd vaccines have strong protective effects on vaccinated individuals (11, 12). The first dose of BNT vaccination has resulted in a 91% reduction in COVID-19 admissions, and ChAd vaccination has induced an 88% reduction. After two vaccination doses, clinical trials for the licensed vaccines have demonstrated 95% efficacy for BNT and 70% efficacy for ChAd against symptomatic diseases (13, 14). BNT can induce high-peak anti-spike IgG titers, and ChAd-induced antibody levels fall slowly (12). However, heterologous vaccines offer higher protection than homologous vaccines. A study showed that a heterologous ChAd–BNT vaccination regimen provided stronger protective immunity than homologous BNT–BNT (15). Another study found that ChAd–BNT heterologous vaccines exhibited significantly stronger immune responses, including the production of stronger cellular and antibody responses, than ChAd–ChAd homologous vaccines(16). Single-cell sequencing (scRNA-seq) technology can measure gene expression on a transcriptome-wide scale (17). In the COVID-19 pandemic, this method has been widely used in revealing the characteristic immune responses of the different immune cells of patients with COVID-19 (18) or recipients of COVID-19 vaccines (19). In addition, a recent study used scRNA-seq to assess the protective capacity of different COVID-19 vaccines (20). However, the molecular mechanisms of differential immune responses induced by heterologous vaccines remain unclear.

COVID-19 vaccination enables recipients to generate a suitable immune response against severe COVID-19 or SARS-CoV-2 infection. Specifically, COVID-19 vaccination can elicit T cell responses (cellular immunity) and B cell responses (antibody immunity) (21). The components of cellular immune responses are CD8^+^ cytotoxic T cells, which kill virus-infected cells with the help of perforin and granzyme and retard and stop infections. CD4^+^ helper T cells activate B cells to produce antibodies specific to antigens. Activated B cells then produce plasma cells and memory B cells, which respond to antigens upon reinfection (22).

In our study, we worked on the immunological effects of different COVID-19 vaccine combination strategies. Blood single cell data on gene expression differences caused by different vaccine strategies were obtained from Gene Expression Omnibus (GEO), and we focused on the gene expression of lymphocytes 7 days after a booster injection. Samples were divided into three groups: homologous BNT–BNT, ChAd–ChAd and heterologous ChAd–BNT according to different prime-boost vaccination strategies. According to the great success of machine learning methods in medicine (23–28), several of them were integrated into a computational framework in this study to identify differences in gene expression induced by vaccines administered with different vaccination strategies. First, the data was investigated by five feature raking algorithms: max-relevance and min-redundancy (mRMR) (29), Monte Carlo feature selection (MCFS) (30), least absolute shrinkage and selection operator (LASSO) (31), light gradient-boosting machine (LightGBM) (32) and permutation feature importance (PFI) (33). Five gene lists were obtained. Then, these lists were subject to incremental feature selection (IFS) (34) method, containing two classification algorithms (decision tree (35) and random forest (36)). After such process, important genes (e.g., *PLCG2*, *B2M*, *JUN*, etc.) and classification rules, indicating different expression patterns for volunteers vaccinated with three different strategies, were accessed. The genes and rules may be useful in discovering vaccination strategies with enhanced protection and long durations, thus providing guidance for prime-boost vaccination.

## 2 Materials and methods

### 2.1 Data

The scRNA-seq profiles of volunteers vaccinated with heterologous ChAd–BNT vaccinations or homologous two ChAd or two BNT doses were derived 7 days after vaccine administration from the GEO database under accession number GSE201534 (37). We mapped the scRNA-seq data to Azimuth datasets, which are well-curated and annotated referenced datasets, and extracted three types of immune cells as the subjects of our analysis, including 3654 B cells, 8212 CD4^+^ T cells, and 5608 CD8^+^ T cells. Each cell was represented by expression levels on 36 601 genes, which were deemed as features in this analysis. Each type of immune cell was classified into three classes according to the original sample as homologous BNT–BNT, homologous ChAd–ChAd, and heterologous ChAd–BNT. The detailed number of each class is provided in **Table 1**.

**Table 1:** Number of cells in each class for three cell types.

Class Cell type	BNT-BNT	ChAd-BNT	ChAd-ChAd
B cell	499	2266	889
CD4^+^ T cell	1148	3335	3729
CD8^+^ T cell	1995	2711	902

### 2.2 Feature ranking algorithms

To date, lots of feature analysis algorithms have been proposed in computer science. Several of them assess the importance of features by ranking them in one list. However, each algorithm has its own advantages and disadvantages. The application of one algorithm to the profiles mentioned in Section 2.1 may produce bias. One algorithm can only mine a part of essential information from the profiles. To obtain essential information as complete as possible, five feature ranking algorithms were employed in this study, which were briefly described as below.

#### 2.2.1 Max-relevance and min-redundancy

The mRMR is a widely used method for assessing the importance of features and often used in gene expression profiling for screening genes with specific biological significance (29, 38, 39). It generates a list to reflect the importance of features. Initially, it is empty. mRMR repeatedly selects a feature from the rest features, which has maximum relevance with respect to a target variable and minimum redundancy with respect to features selected during previous iterations. Relevance and redundancy are measured according to mutual information, which is expressed by the following equation:


, (1) $$MI\left(x,y\right)=\iint^{}p\left(x,y\right)\log\frac{p\left(x,y\right)}{p\left(x\right)p\left(y\right)}dxdy$$

where *p*(*x*) and *p*(*y*) stand for the marginal probabilistic densities of *x* and *y*, respectively, *p*(*x*,*y*) stands for the joint probabilistic density of *x* and *y*. When all features are in the list, the procedures stop. In the present study, we utilized the mRMR program from Peng’s lab (http://home.penglab.com/proj/mRMR/) and ran the analysis by using the default settings.

#### 2.2.2 Monte Carlo feature selection

The MCFS is a DT-based feature importance evaluation algorithm and commonly used to process biological data (30, 40, 41). In MCFS, *m* features are randomly selected to comprise a feature subset. On such subset, *t* DTs are constructed using different randomly selected training samples. Above procedure executes *s* times, thereby generating *s*×*t* trees. A feature’s relative importance (RI), as measured by how many times it has been selected by these trees and how much it contributes to prediction of the trees, was estimated as follows:


, (2) $$RI_{g}=\;\sum_{\tau =1}^{s\times t}{\left(wAcc\right)}^{u}\sum_{n_{g}\left(\tau \right)}IG\left(n_{g}\left(\tau \right)\right){\left(\frac{no.in\;n_{g}\left(\tau \right)}{no.in\;\tau }\right)}^{v}$$

where *wAcc* is the weighted accuracy, *IG*(*n*_*g*_(*τ*)) is the information gain (IG) of node *n*_*g*_(*τ*) , ( *no*.*in* *n*_*g*_(*τ*))  is the number of samples in node *n*_*g*_(*τ*) , and (*no*.*in* *τ*) is the sample sizes in the tree root; *u* and *v* are two settled positive integers. After each feature is assigned a RI score, features are sorted in a list with decreasing order of their RI scores. In the present study, the MCFS program was retrieved from http://www.ipipan.eu/staff/m.draminski/mcfs.html. It was performed using its default parameters.

#### 2.2.3 Least absolute shrinkage and selection operator

A penalty function that selectively eliminates features was created by applying a high penalty to features with high coefficients and using an L1 paradigm in LASSO. This practice has the effect of actually forcing some coefficients to become zero, which effectively performs feature selection by removing features from models (31). As a result, the coefficients of features can be used to rank features. This study used the LASSO program collected in Scikit-learn (42). Default parameters were used to execute such program.

#### 2.2.4 Light gradient-boosting machine

The LightGBM is a gradient-boosting framework based on DTs, which can increase the efficiency of models and reduce memory usage (32). As a measure of feature importance for prediction, the LightGBM counts the total number of times (i.e., *T* *Split* ) that each feature is used in trees and the benefits (i.e., *T* *Gain* ) that a feature receives from being used for splitting in all DTs.


, (3) $$T\;Split=\;\sum_{t=1}^{K}Split_{t}$$

, (4) $$T\;Gain=\;\sum_{t=1}^{K}Gain_{t}$$

where *K* is the number of DTs generated by *K* iterations. Here, we used the setting of split as a metric in measuring the importance of features. Features are ranked in a list with decreasing order of their splits. The LightGBM program used in this study was sourced from https://lightgbm.readthedocs.io/en/latest/. For convenience, it was executed with default parameters.

#### 2.2.5 Permutation feature importance

Permutation feature importance (PFI) was first introduced in 2001 by Breiman for RFs and was later extended to fitted estimators by Fisher, Rudin, and Dominici (33, 36). If a feature is more important, after its values are shuffled, prediction error will increase. A feature is considered unimportant if shuffling its values does not increase prediction error. Its computations include the following steps:

1. The training model is denoted as *f* ; the feature matrix, as *X* ; target variable, as *y* ; and the error measure, as *L*(*y*,*f*) .

2. Given a dataset *X* , its baseline prediction error is calculated as *e*_*base*_=*L*(*y*,*f*(*X*)) .

3. Given a feature *j*∈{1,…,*J*} for each repetition *k*∈{1,…,*K*}

a) Randomly shuffle feature *j* , and generate a permuted version of feature matrix *X*_*perm*_ ;

b) Estimate the prediction error *e*_*j*,*k*_=*L*(*y*,*f*(*X*_*perm*_)) based on the permuted data *X*_*perm*_ ;

c) Calculate differences between baseline score and the shuffled dataset score as the feature importance *I*_*j*,*k*_=*e*_*j*,*k*_/*e*_*base*_ .

4. Calculate the mean score of the feature importance $I_{j}=\frac{1}{K}\sum_{k=1}^{K}I_{j,k}$ .

5. Sort the features based on *I*_*j*_ .

Here, we used the PFI program downloaded from scikit-learn (42), which was performed with default parameters.

The profiles mentioned in Section 2.1 were fed into above five feature ranking algorithms. Each algorithm yielded a gene list. For an easy description, these lists were called mRMR, MCFS, LASSO, LightGBM and PFI gene lists, respectively.

### 2.3 Incremental feature selection

As stated in Section 2.2, five gene lists can be obtained using five feature ranking algorithms. The best feature subset for classification can be extracted from each list. The IFS method was introduced to complete this task. IFS is a popular approach for finding the optimal feature subset for classification using a supervised classification algorithm (34, 43, 44). The IFS method was applied to each gene list. Its procedures can be broken down into the following main steps: (1) From the gene list, several gene subsets were constructed by repeatedly adding ten features, i.e., the first subset contained the first ten genes, the second subset included the top twenty features, and so forth. (2) On each gene subset, one classifier was built using genes in this subset and it was evaluated by 10-fold cross-validation (45). (3) The feature set and classifier with the best classification performance are referred to as the optimal feature subset and classifier, respectively.

### 2.4 Synthetic minority oversampling technique

As listed in **Table 1**, the profile for each cell type is imbalanced. The classifier directly built on such profile may produce bias. The profile must be processed first to reduce the influence of imbalanced problem. This study adopted the synthetic minority oversampling technique (SMOTE), which is a data augmentation technique for minorities (46–48). Beginning with samples that are close to a randomly selected sample in a feature space, SMOTE creates a new sample along the line it draws between two samples. Specifically, a random sample from a minority class is initially determined. The *k* nearest neighbors in the same class are then observed for that sample. A synthetic sample is built at a randomly selected place in a feature space between the sample and its randomly selected neighbor. For each class except the largest class, SMOTE repeatedly generated several new samples until this class contained samples as many as those in the largest class. The SMOTE algorithm in this study was implemented *via* python.

### 2.5 Classification algorithm

Classification algorithm is necessary to execute IFS method. Here, two algorithms (DT (35) and RF (36)) were employed. They have wide applications in dealing with medical and biological problems (49–55).

#### 2.5.1 Decision tree

DTs are basic classification and regression methods with tree-like structures (35). A DT model represents the classification and discrimination of data as a tree-like structure with nodes and directed edges. When a rule is built for each path of a DT from the root node to the leaf node, each internal node corresponds to the rule’s condition, and a leaf node displays the outcome of an associated rule. Thus, a DT can be deemed as a collection of if-then rules. To implement DT, we employed the CART method and the scikit-learn package (42), with Gini coefficients serving as the IG.

#### 2.5.2 Random forest

RF is an ensemble method that adopts DT as the basic unit (36). In the concentration of a forest, trees are created several times using randomly selected features and samples. The sample is predicted by aggregating the predictions of all DTs. The RF package from Python’s scikit-learn module was employed in this study for building RF classifiers in the IFS method.

### 2.6 Performance evaluation

The weighted F1 was used in mainly evaluating the performance of classifiers that were constructed in IFS method. To calculate such measurement, the F1-measure for each class should be computed first, as follows:


, (5) $$Precision_{i}=\frac{TP_{i}}{TP_{i}+FP_{i}}$$

, (6) $$Recall_{i}=\frac{TP_{i}}{TP_{i}+FN_{i}}$$

, (7) $$F1-measure_{i}=\frac{2\times Precision_{i}\times Recall_{i}}{Precision_{i}+Recall_{i}}$$

where  *i* denotes the index of one class, *TP_i_*, *FP_i_* and *FN_i_* denote true positive, false positive and false negative for the *i*-th class, respectively. Then, the weighted F1 can be computed by


, (8) $$Weighted\;F1=\sum_{i=1}^{L}w_{i}\times F1-measure_{i}$$

where *w*_*i*_  denotes the proportion of samples in the *i*-th class to all samples, *L* denotes the total number of classes.

In addition, the macro F1, prediction accuracy (ACC) and Matthew correlation coefficients (MCC) (56) were also employed in this study to fully display the performance of all classifiers. Macro F1 is similar to weighted F1, which is the direct average of all F1-measure values. ACC is one of the most widely used measurements, which is defined as the proportion of correctly predicted samples to all samples. MCC is a balanced measurement. When the dataset is imbalanced, it is much more accurate than ACC. It can be computed by


, (9) $$MCC=\frac{cov\left(X,Y\right)}{\sqrt{cov\left(X,X\right)\times cov\left(Y,Y\right)}}$$

where *X* and *Y* are two matrices, storing the true and predicted classes of all samples, respectively, cov(*X*,*Y*) stands for the covariance of *X* and *Y*.

## 3 Results

**Figure 1:**
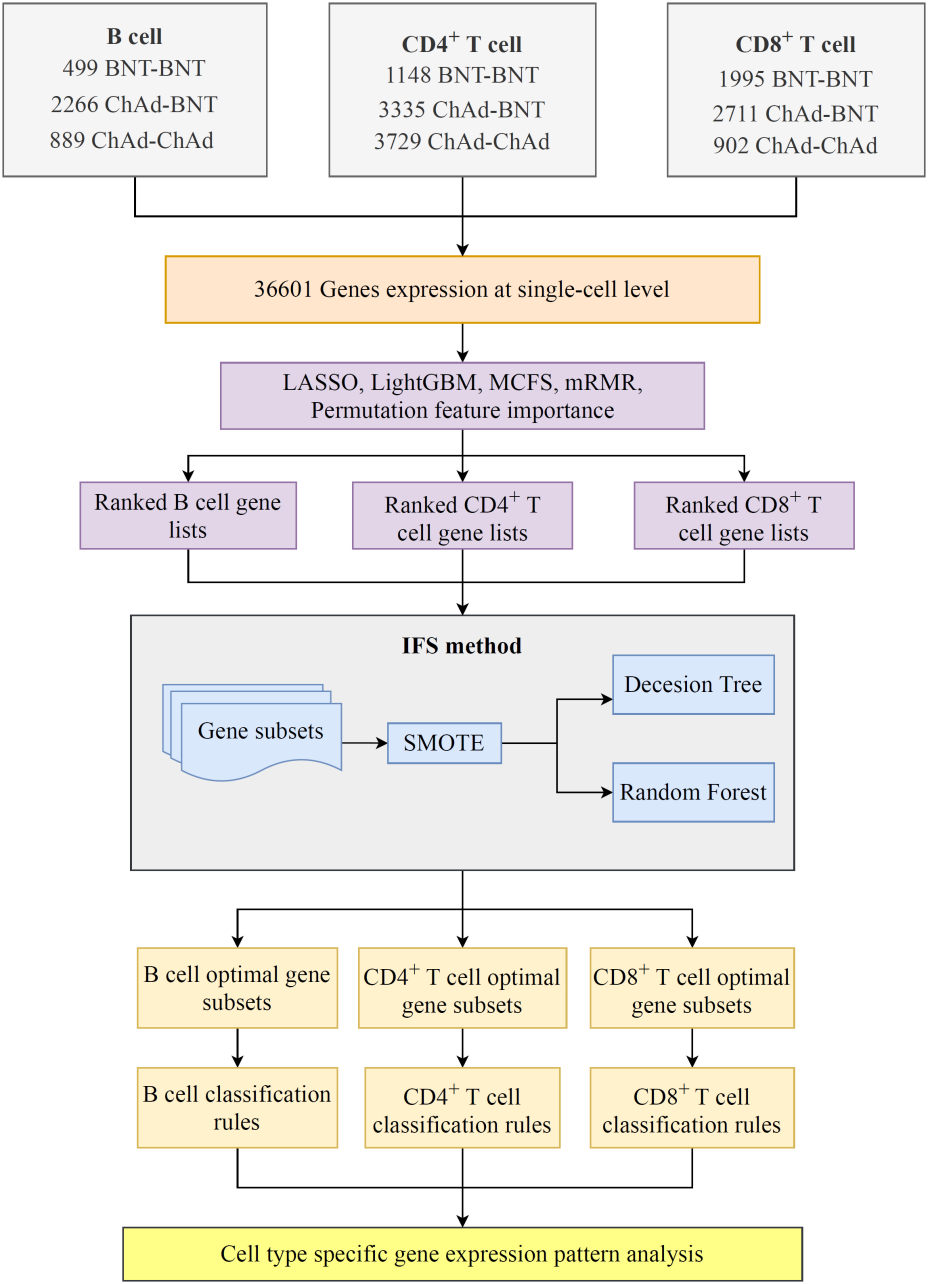
Flowchart of the computational framework that integrates multiple feature selection algorithms and classification algorithms. The single-cell profiles of COVID-19 includes B, CD4^+^ T, and CD8^+^ T cells, each of which has three vaccination states, namely, BNT–BNT, ChAd–BNT, and ChAd–ChAd. On each cell type, a set of gene lists were obtained using five feature ranking algorithms: LASSO, LightGBM, mRMR, MCFS, and permutation feature importance (PFI). Subsequently, the optimal classifiers and the corresponding optimal feature subsets on each gene list were obtained using the incremental feature selection (IFS) method. Finally, the classification rules were mined by each optimal decision tree (DT) classifier.

In the current work, we employed several efficient feature selection methods and classification algorithms to design a computational framework for mining significant genes and rules in various cell types, which can determine the efficacy of homologous and heterologous COVID-19 vaccines. The overall computational framework is shown in **Figure 1**. The results associated with each step of the computation process are described below.

### 3.1 Feature ranking results

The current study included three cell types with a total of 17 474 cells and 36 601 genes. As shown in **Supplementary Table S1**, genes were sorted for each cell type using five feature ranking algorithms to provide a set of feature lists (mRMR, MCFS, LASSO, LightGBM and PFI gene lists). The feature lists for B, CD4^+^ T, and CD8^+^ T cells would be entered into the IFS method to determine the optimal features.

### 3.2 Results of IFS method with RF and DT algorithms

**Figure 2:**
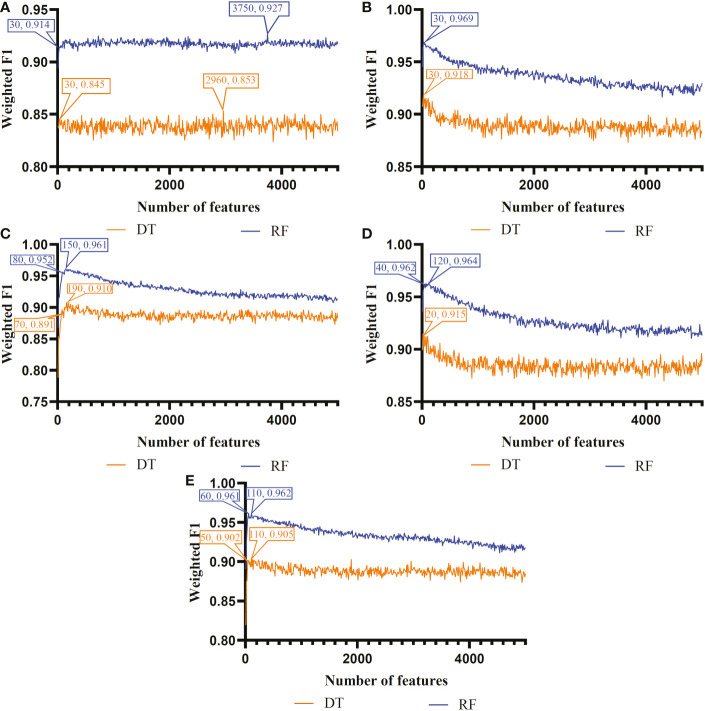
Incremental feature selection (IFS) curves of two classification algorithms in B cells. **(A)** IFS results obtained based on the LASSO gene list. **(B)** IFS results obtained based on the LightGBM gene list. **(C)** IFS results obtained based on the mRMR gene list. **(D)** IFS results obtained based on the MCFS gene list. **(E)** IFS results obtained based on the PFI gene list.

**Figure 3:**
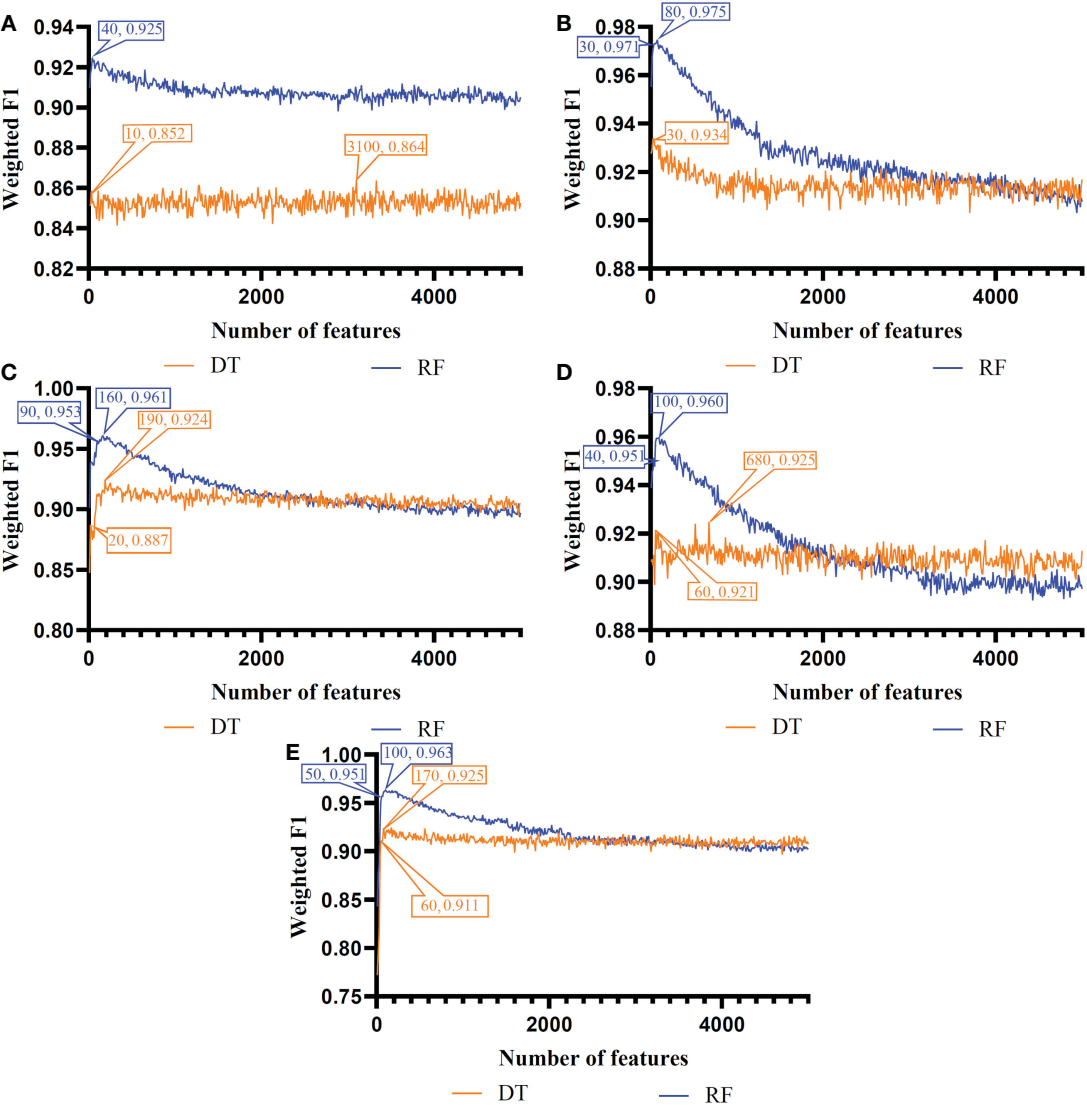
Incremental feature selection (IFS) curves of two classification algorithms in CD8^+^ T cells. **(A)** IFS results obtained based on the LASSO gene list. **(B)** IFS results obtained based on the LightGBM gene list. **(C)** IFS results obtained based on the mRMR gene list. **(D)** IFS results obtained based on the MCFS gene list. **(E)** IFS results obtained based on the PFI gene list.

**Figure 4:**
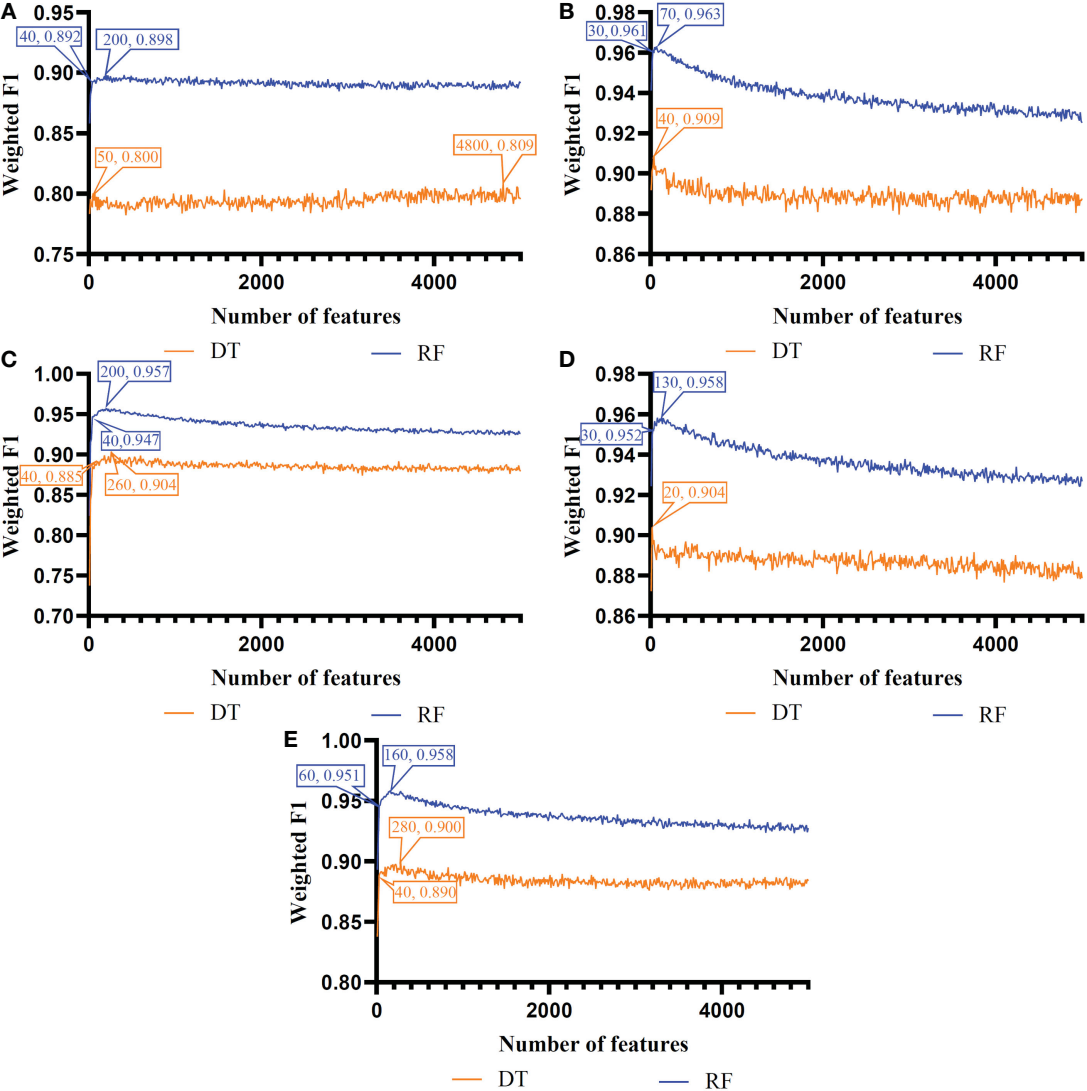
Incremental feature selection (IFS) curves of two classification algorithms in CD4^+^ T cells. **(A)** IFS results obtained based on the LASSO gene list. **(B)** IFS results obtained based on the LightGBM gene list. **(C)** IFS results obtained based on the mRMR gene list. **(D)** IFS results obtained based on the MCFS gene list. **(E)** IFS results obtained based on the PFI gene list.

The IFS method was used in combination with RF and DT to determine the optimal features and construct the best classifiers for each cell type. The mRMR, MCFS, LASSO, LightGBM and PFI gene lists were used in this procedure. Considering the huge number of gene features, only top 5000 features in each list were considered and feature subsets were constructed using ten as the step. Thus, 500 feature subsets were generated from each list. DT and RF classifiers were built using features in each subset and evaluated by 10-fold cross-validation. In the 10-fold cross-validation, the SMOTE was utilized in creating samples for minor classes in the training dataset, which addressed the problem of sample imbalance. Weighted F1 was used in assessing the performance of all classifiers. The detailed results of the IFS method are shown in **Supplementary Table S2**. With weighted F1 on the Y-axis and the number of features on the X-axis, **Figures 2**–**4** depict the IFS curves of DT and RF in B, CD4^+^ T, and CD8^+^ T cells.

For B cell, the IFS curves of DT and RF on five gene lists are shown in **Figure 2**. On the LASSO gene list, DT and RF yielded the highest weighted F1 values of 0.853 and 0.927, respectively, when top 2960 and 3750 features were adopted. These features were deemed as the optimal features for DT and RF identified by LASSO. With such features, the optimal DT and RF classifiers were built. Under the similar operation, the optimal DT and RF classifiers on other four gene lists can be set up. In detail, the optimal DT and RF classifiers on LightGBM gene list used the top 30 and 30 features, respectively, yielding the weighted F1 values of 0.918 and 0.969. On the MCFS gene list, such two optimal classifiers employed the top 190 and 150 features, and their weighted F1 values were 0.910 and 0.961, respectively. For the mRMR gene list, top 20 and 120 features were used to build the optimal DT and RF classifiers, generating the weighted F1 values of 0.915 and 0.964, respectively. For the last PFI gene list, the two optimal classifiers were set up using top 110 and 110 features, producing the weighted F1 values of 0.905 and 0.962, respectively. The detailed performance of above optimal classifiers, including F1-measure on three classes, ACC, MCC, macro F1 and weighted F1, are listed in **Table 2**. All these classifiers were quite good with weighted F1 around 0.900. Obviously, given the classification algorithm (DT or RF), the optimal classifier on LightGBM gene list always provided the best performance.

**Table 2:** Performance of key classifiers in B cell.

Feature ranking algorithm	Classification algorithm	Number of features	BNT-BNT	ChAd-BNT	ChAd-ChAd	ACC	MCC	Macro F1	Weighted F1
LASSO	DT	2960	0.974	0.880	0.717	0.851	0.730	0.857	0.853
	DT^*^	30	0.972	0.870	0.713	0.841	0.722	0.851	0.845
	RF	3750	0.989	0.943	0.850	0.927	0.864	0.927	0.927
	RF^*^	30	0.992	0.928	0.833	0.912	0.843	0.918	0.914
LightGBM	DT	30	0.982	0.934	0.842	0.918	0.850	0.919	0.918
	RF	30	0.999	0.974	0.937	0.969	0.942	0.970	0.969
MCFS	DT	190	0.980	0.929	0.822	0.909	0.833	0.910	0.910
	DT^*^	70	0.977	0.910	0.797	0.889	0.802	0.894	0.891
	RF	150	0.998	0.969	0.921	0.961	0.928	0.963	0.961
	RF^*^	80	0.996	0.961	0.904	0.952	0.911	0.954	0.952
mRMR	DT	20	0.973	0.933	0.837	0.914	0.844	0.914	0.915
	RF	120	0.994	0.971	0.928	0.964	0.932	0.964	0.964
	RF^*^	40	0.998	0.969	0.923	0.962	0.929	0.963	0.962
PFI	DT	110	0.972	0.924	0.820	0.904	0.825	0.905	0.905
	DT^*^	50	0.961	0.923	0.816	0.901	0.819	0.900	0.902
	RF	110	1.000	0.969	0.921	0.961	0.928	0.963	0.962
	RF^*^	60	0.997	0.968	0.920	0.960	0.927	0.962	0.961

*: Feasible classifiers with much less features and a little lower performance than optimal classifiers.

For CD4^+^ T cell, the optimal DT and RF classifiers on each gene list can be extracted from **Figure 4**. On LASSO gene list, top 4800 and 200 features were used. Optimal feature numbers on other four gene lists were 40 and 70 (LightGBM gene list), 260 and 200 (MCFS gene list), 20 and 130 (mRMR gene list), 280 and 160 (PFI gene list). **Table 3** lists the detailed performance of these optimal classifiers. These classifiers also provided high performance, similar to those for B cell. Similarly, on the LightGBM gene list, the DT/RF optimal classifier always provided the best performance.

**Table 3:** Performance of key classifiers in CD4^+^ T cell.

Feature ranking algorithm	Classification algorithm	Number of features	BNT-BNT	ChAd-BNT	ChAd-ChAd	ACC	MCC	Macro F1	Weighted F1
LASSO	DT	4800	0.967	0.773	0.792	0.809	0.687	0.844	0.809
	DT^*^	50	0.968	0.765	0.780	0.800	0.673	0.838	0.800
	RF	200	0.996	0.874	0.890	0.898	0.833	0.920	0.898
	RF^*^	40	0.993	0.868	0.883	0.892	0.823	0.915	0.892
LightGBM	DT	40	0.981	0.891	0.903	0.909	0.851	0.925	0.909
	RF	70	0.999	0.954	0.960	0.963	0.939	0.971	0.963
	RF^*^	30	1.000	0.952	0.958	0.961	0.936	0.970	0.961
MCFS	DT	260	0.973	0.887	0.898	0.904	0.842	0.919	0.904
	DT^*^	40	0.972	0.864	0.876	0.885	0.811	0.904	0.885
	RF	200	0.997	0.947	0.954	0.957	0.929	0.966	0.957
	RF^*^	40	0.994	0.935	0.943	0.947	0.913	0.957	0.947
mRMR	DT	20	0.985	0.884	0.897	0.904	0.842	0.922	0.904
	RF	130	0.997	0.949	0.955	0.958	0.932	0.967	0.958
	RF^*^	30	0.997	0.942	0.948	0.952	0.922	0.962	0.952
PFI	DT	280	0.974	0.881	0.895	0.900	0.836	0.916	0.900
	DT^*^	40	0.969	0.871	0.884	0.890	0.820	0.908	0.890
	RF	160	0.998	0.949	0.955	0.958	0.932	0.967	0.958
	RF^*^	60	0.985	0.942	0.949	0.951	0.920	0.959	0.951

*: Feasible classifiers with much less features and a little lower performance than optimal classifiers.

As for the CD8^+^ T cell, we also built the optimal DT and RF classifiers by applying IFS method on five gene lists. According to **Figure 3**, their optimal feature numbers were 3100 and 40 (LASSO gene list), 30 and 80 (LightGBM gene list), 190 and 160 (MCFS gene list), 680 and 100 (mRMR gene list), 170 and 100 (PFI gene list). The detailed performance of these classifiers is listed in **Table 4**. Similar to the optimal classifiers for B and CD4^+^ T cells, these classifiers also yielded high performance. Again, the optimal DT/RF classifier on LightGBM gene list generated the best performance.

**Table 4:** Performance of key classifiers in CD8^+^ T cell.

Feature ranking algorithm	Classification algorithm	Number of features	BNT-BNT	ChAd-BNT	ChAd-ChAd	ACC	MCC	Macro F1	Weighted F1
LASSO	DT	3100	0.987	0.856	0.617	0.860	0.777	0.820	0.864
	DT^*^	10	0.987	0.833	0.608	0.843	0.760	0.809	0.852
	RF	40	0.997	0.919	0.781	0.923	0.877	0.899	0.925
LightGBM	DT	30	0.990	0.932	0.813	0.933	0.892	0.912	0.934
	RF	80	0.997	0.974	0.926	0.975	0.959	0.966	0.975
	RF^*^	30	0.997	0.970	0.915	0.971	0.952	0.961	0.971
MCFS	DT	190	0.988	0.922	0.785	0.922	0.875	0.899	0.924
	DT^*^	20	0.983	0.882	0.690	0.882	0.816	0.852	0.887
	RF	160	0.997	0.959	0.886	0.961	0.936	0.947	0.961
	RF^*^	90	0.995	0.952	0.865	0.953	0.924	0.937	0.953
mRMR	DT	680	0.992	0.922	0.786	0.924	0.877	0.900	0.925
	DT^*^	60	0.987	0.918	0.788	0.920	0.872	0.897	0.921
	RF	100	0.996	0.958	0.884	0.959	0.934	0.946	0.960
	RF^*^	40	0.997	0.948	0.858	0.950	0.921	0.935	0.951
PFI	DT	170	0.985	0.924	0.791	0.924	0.877	0.900	0.925
	DT^*^	60	0.982	0.909	0.759	0.909	0.855	0.884	0.911
	RF	100	0.997	0.962	0.892	0.963	0.939	0.950	0.963
	RF^*^	50	0.995	0.949	0.862	0.950	0.920	0.935	0.951

*: Feasible classifiers with much less features and a little lower performance than optimal classifiers.

**Figure 5:**
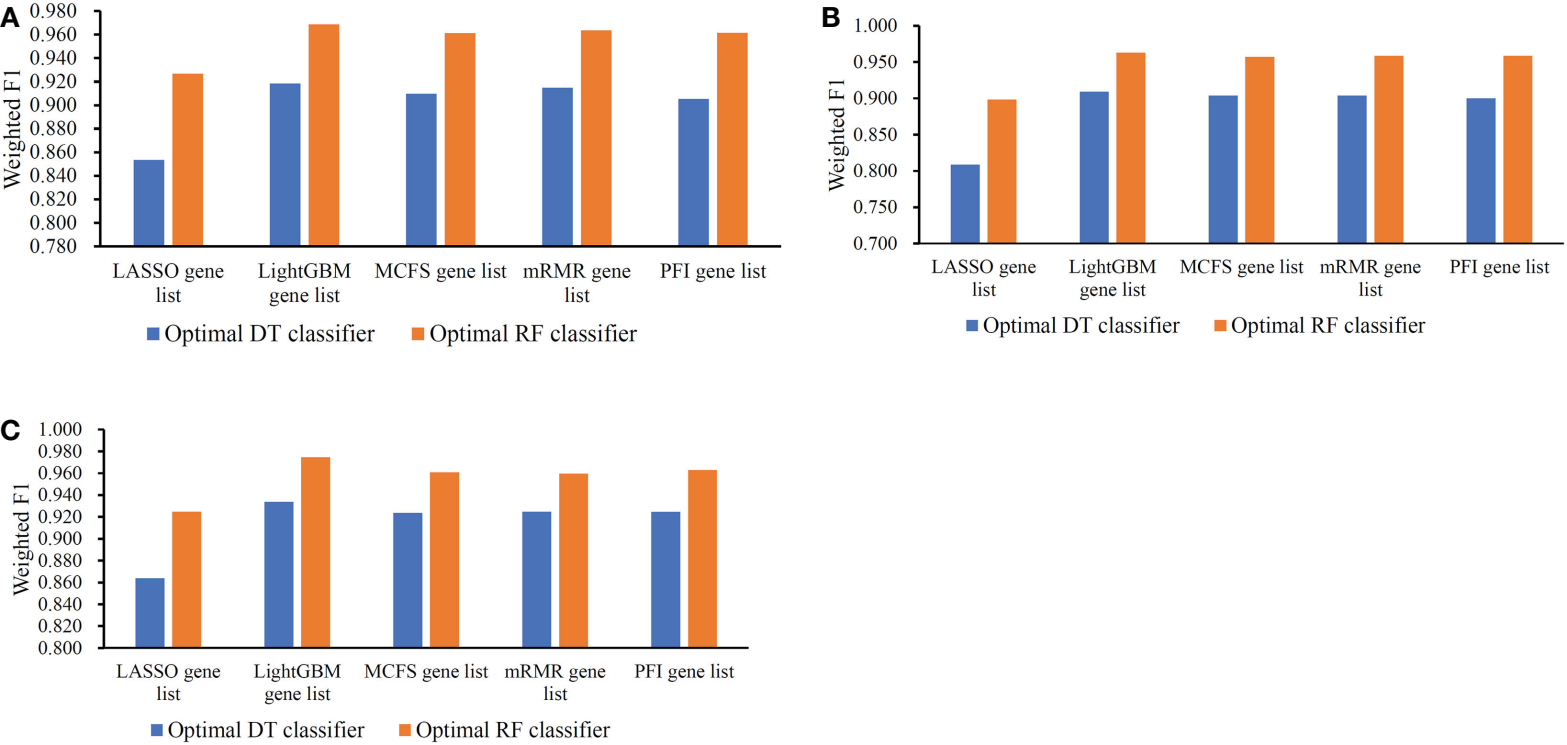
Bar chart to show weighted F1 yielded by the optimal classifiers on different gene lists for three cell types. **(A)** Bar chart for B cell. **(B)** Bar chart for CD4^+^ T cell. **(C)** Bar chart for CD8^+^ T cell.

Based on the performance of optimal DT and RF classifiers for three cell types (**Figures 2**–**4** and **Tables 2**–**4**), RF classifiers were always better than DT classifiers. For B cell, the optimal RF classifier on LightGBM gene list was best, which used the top 30 features in the LightGBM gene list. For other two cell types, same results can be obtained, i.e., the optimal RF classifier on LightGBM gene list was better than other optimal classifiers. Top 70 (CD4^+^ T cell) and 80 (CD8^+^ T cell) features in corresponding LightGBM gene lists were used. Five feature ranking algorithms were employed in this study, it is necessary to investigate their utilities in analyzing the scRNA-seq profiles. The weighted F1 values of the optimal DT/RF classifiers for three cell types are illustrated in **Figure 5**. It was interesting that the trendies of weighted F1 values yielded by the optimal DT/RF classifier on different gene lists were quite similar for three cell types. On LightGBM gene lists, the optimal classifiers were always best, as mentioned in above paragraphs, the optimal classifiers on LASSO gene list were evidently inferior to the optimal classifiers on other four gene lists, the performance of the optimal classifiers on MCFS, mRMR and PFI gene lists was quite close. It was indicated that LightGBM may be the best algorithm to analyze the profiles, the abilities of MCFS, mRMR and PFI were almost equal and LASSO was weaker than others.

**Figure 6:**
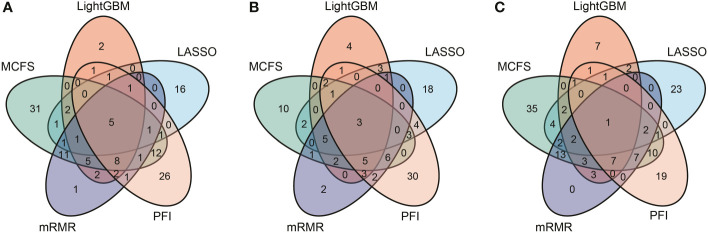
Venn results of five essential feature subsets identified by five feature ranking algorithms in three immune cell types. Genes found in multiple overlapping circles indicate that they were highly ranked in multiple ranking algorithms and were more likely to differ in homologous and heterologous vaccine immune responses. **(A)** Venn results for B cell. **(B)** Venn results for CD4+ T cell. **(C)** Venn results for CD8+ T cell.

According to **Figures 2**–**4**, some optimal DT or RF classifiers on different gene lists used lots of features. The efficiencies of these classifiers were not high enough to process the large-scale data. By checking the IFS results in **Supplementary Table S2**, the feasible DT or RF classifiers were built for some optimal DT or RF classifiers that adopted lots of features. For example, the optimal DT classifier on LASSO gene list for B cell used the top 2960 features. However, the DT classifier with top 30 features yielded the weighted F1 of 0.845, a little lower than that of the optimal DT classifier (0.853). Much less features sharply increased the efficiency but the utility was limited dropped. Thus, we named it as the feasible DT classifier. In **Tables 2**–**4**, the performance of all feasible classifiers is listed (see rows marked by “*”). Clearly, their performance was a little lower than the corresponding optimal classifiers. Notably, it was not necessary to identify the feasible classifiers for the optimal classifiers that adopted a small quantity of features. Multiple feature ranking algorithms were employed in this study to analyze the profiles on three cell types. They may all give contributions to mine essential information from the profiles. In view of this, the features used to construct feasible RF classifiers (if available) or optimal RF classifiers on five gene lists for each cell type were picked up. Five feature sets were obtained accordingly for each cell type. The Venn diagram, as illustrated in **Figure 6**, shows the relationships between these feature sets. Some features (genes) belonged to multiple sets, meaning that multiple feature ranking algorithms identified them as essential genes. The detailed overlapped results are provided in **Supplementary Table S3**. In Section 4, we would focus on the biological significance of some overlapped genes.

### 3.3 Classification rules created by the optimal DT classifier

For each cell type and each gene list, the optimal DT classifier was inferior to the optimal RF classifier. However, DT has a great merit. As it is a white-box algorithm, i.e., the classification procedures are completely open, it provides more possible to uncover hidden information that human can understand. Here, we used each optimal DT classifier to generate classification rules, which are available in **Supplementary Table S4**. The number of rules based on each gene list for each cell type is listed in **Table 5**. Each rule incorporated many gene features and specified requirements for their quantitative expression, revealing different gene expression patterns for three different vaccination strategies in three cell types. Some important rules would be discussed in detail in Section 4.

**Table 5:** Breakdown of rules yielded by decision tree on different gene lists for each cell type.

Cell type	Gene list	Number of rules			
		BNT-BNT	ChAd-BNT	ChAd-ChAd	Total
B cell	LASSO gene list	13	93	138	244
	LightGBM gene list	15	88	82	185
	MCFS gene list	11	77	75	163
	mRMR gene list	16	106	87	209
	PFI gene list	14	83	70	167
CD4^+^ T Cell	LASSO gene list	37	297	327	661
	LightGBM gene list	28	189	178	395
	MCFS gene list	27	162	163	352
	mRMR gene list	25	205	202	432
	PFI gene list	27	162	166	355
CD8^+^ T Cell	LASSO gene list	18	141	178	337
	LightGBM gene list	18	117	110	245
	MCFS gene list	17	109	104	230
	mRMR gene list	22	92	90	204
	PFI gene list	21	101	95	217

## 4 Discussion

### 4.1 Analysis of gene features in lymphocytes associated with COVID-19 vaccination

On the basis of our computational framework, a set of important genes were identified, which were differentially expressed in B and T cells and facilitated distinction among the immunological effects of different prime-boost vaccinations. As shown in **Figure 6**, some genes were identified by multiple feature ranking algorithms. These genes may be highly related to different biological activities in lymphocytes that perform immune functions after vaccination or natural infection. Here, we selected five genes in B, CD4^+^ T, and CD8^+^ T cells for detailed analysis, which are listed in **Table 6**.

**Table 6:** Top five genes identified by the computational framework in lymphocytes.

Cell Type	Ensembl ID	Gene symbol	Description
B Cell	ENSG00000197943	PLCG2	phospholipase C gamma 2
	ENSG00000237541	HLA-DQA2	major histocompatibility complex, class II, DQ alpha 2
	ENSG00000147403	RPL10	ribosomal protein L10
	ENSG00000198938	MT-CO3	mitochondrially encoded cytochrome c oxidase III
	ENSG00000198712	MT-CO2	mitochondrially encoded cytochrome c oxidase II
CD4^+^ T Cell	ENSG00000198938	MT-CO3	mitochondrially encoded cytochrome c oxidase III
	ENSG00000166710	B2M	beta-2-microglobulin
	ENSG00000147403	RPL10	ribosomal protein L10
	ENSG00000198034	RPS4X	ribosomal protein S4 X-linked
	ENSG00000149273	RPS3	ribosomal protein S3
CD8^+^ T Cell	ENSG00000269028	MTRNR2L12	MT-RNR2 like 12
	ENSG00000197943	PLCG2	phospholipase C gamma 2
	ENSG00000177606	JUN	Jun proto-oncogene
	ENSG00000213741	RPS29	ribosomal protein S29
	ENSG00000229807	XIST	X inactive specific transcript

#### 4.1.1 Qualitative features in B cells

The first identified feature gene was *PLCG2* (ENSG00000197943). PLCG2 is involved in B cell receptor signaling pathway (57–59) and B cell differentiation (58). Mutations in *PLCG2* can impaire B cell memory and antibody production (60). In addition, the protein encoded by the *PLCG2* gene, phospholipase Cγ2, plays an important role in the transmembrane transduction of immune signals (61, 62). In summary, *PLCG2* is closely related to B cells, and differential expression of *PLCG2* is important to the B cell immune pathway. Recent publications have provided evidence of the differential expression of *PLCG2* after COVID-19 vaccination. Although no direct evidence of COVID-19 vaccination has been reported, changes in *PLCG2* expression after infection with SARS-CoV-2 partially demonstrate the effectiveness of *PLCG2* as a feature. In 2022, a study found that *PLCG2* was upregulated as an infection-associated gene in the kidneys of patients with COVID-19 (63). Another 2022 study found no elevated anti-S1 IgA levels in a subject carrying a mutation in the *PLCG2* gene after a booster dose of BNT vaccine, suggesting a role of *PLCG2* after COVID-19 vaccination (64). Therefore, *PLCG2* gene expression may be altered after vaccination, and PLCG2 in B cells can be used as an effective feature.

The next identified feature was *HLA*–*DQA2* (ENSG00000237541). *HLA*–*DQA2* gene is involved in immunoglobulin production and immunoglobulin-mediated immune responses (65) and is involved in antigen processing and presentation of exogenous peptide antigens *via* MHC class II (65, 66). Thus, *HLA*–*DQA2* plays an important role in immune response. COVID-19 vaccines may have an impact on how *HLA*–*DQA2* is expressed, though direct evidence following COVID-19 vaccination is limited. In recent year, researchers have shown that *HLA*–*DQA2* gene expression can be changed according to COVID-19 severity and *HLA*–*DQA2* was upregulated in patients with mild COVID-19 and those who recovered (67, 68) and downregulated in patients with severe COVID-19 (66, 67, 69). *HLA*–*DQA2* gene expression may have a trend similar to that in patients with mild COVID-19 and recovered individuals after vaccination. In addition, *HLA*–*DQA2* gene plays an immunological role in B cells as previously mentioned, suggesting altered expression after vaccination. Therefore, *HLA–DQA2* may be altered in expression after COVID-19 vaccination. In conclusion, the expression of *HLA*–*DQA2* in B cells can be a useful feature.

The next identified feature genes were *MT-CO2* (ENSG00000198712) and *MT-CO3* (ENSG00000198938). *MT-CO2* and *MT-CO3* are mitochondrial genes involved in aerobic respiration and ATP synthesis for energy supply (65, 70). Viral infection is known to affect mitochondrial function (71, 72), and mitochondrial genes play a key role in the host immune response (73, 74). *MT-CO2* and *MT-CO3* gene expression may be altered after COVID-19 vaccination, and SARS-CoV-2 infection can alter *MT-CO2* and *MT-CO3* expression. For example, *MT-CO2* and *MT-CO3* are downregulated in severe COVID-19 patients (75, 76). An increased expression of mitochondrial genes was found in SARS-CoV-2-infected lung cells (77) and upregulated expression in alveolar epithelial cells of patients with mild or moderate COVID-19 (78). Furthermore, in 2022, Adamo et al. viewed the *MT-CO2* gene as a gene tag for recovery in COVID-19 patients and considered that increase in its expression indicates improvement in COVID-19 (79), suggesting that immune response induced by COVID-19 vaccination also leads to differential *MT-CO2* and *MT-CO3* gene expression. According to the functions of *MT-CO2* and *MT-CO3* genes discussed above, their expression may be changed after COVID-19 vaccination and may respond to the intensity of the immune response after vaccination. Therefore, *MT-CO2* and *MT-CO3* are potentially useful features.

The last identified gene was *RPL10* (ENSG00000147403). *RPL10* encodes ribosomal protein L10, which promotes ribosome biogenesis and its ability to synthesize proteins (80–82). Thus, immunoglobulins, such as antibodies, as proteins, and *RPL10* in B cells may play a role in immunoglobulin production. Some recent papers have provided evidence of altered *RPL10* gene expression after COVID-19 vaccination. Chang et al. suggested that ribosomal genes, such as *RPL10*, can serve as biomarkers for identifying SAR-CoV-2 infection (83), suggesting the possibility of altered *RPL10* gene expression after vaccination. Similarly, another study found altered expression of ribosomal genes, including *RPL10*, after SARS-CoV-2 infection (84). COVID-19 vaccination produces a large number of antibodies (85–87), and based on the previously postulated contribution of the *RPL10* gene to antibody production by B cells, *RPL10* expression may be altered after vaccination. As a result, *RPL10* gene in B cells can be an effective feature.

#### 4.1.2 Qualitative features in CD4+ T cells

The first identified feature gene was *B2M* (ENSG00000166710), which has a broad role in immune response and is a marker of lymphocyte turnover (88, 89). In T cells, *B2M* is associated with thymic T cell differentiation (90), involved in the positive regulation of T cell activation (65) and is a hub gene for cytokine storm (91). COVID-19 vaccination may cause the differential expression of *B2M* gene. *B2M* is widely recognized as a good biomarker of responses to COVID-19 severity and treatment (92, 93) and is upregulated in the olfactory bulb of patients of COVID-19 (92). SARS-CoV-2 natural infection alters *B2M* expression, partially demonstrating the differential expression of *B2M* after vaccination. Given the previously mentioned important role of *B2M* gene in immune response, immune response induced by a COVID-19 vaccine may alter the expression of *B2M* Thus, *B2M* is a potential feature.

The next identified gene was *MT-CO3* (ENSG00000198938), which is a mitochondrial gene that we have previously discussed as a feature in B cells. Although little has been written about the specific role of *MT-CO3* in CD4^+^ T cells, according to the important role of *MT-CO3* gene in the immune response (94) and its involvement in aerobic respiratory energy supply (65, 70), *MT-CO3* gene may show differential expression after COVID-19 vaccination. Furthermore, SARS-CoV-2 natural infection leads to differential *MT-CO3* gene expression (75–77), further demonstrating that immunological response induced by COVID-19 vaccination may change *MT-CO3* expression. As a result, *MT-CO3* gene in CD4^+^ T cells can be used as an effective feature.

The next identified features were *RPL10* (ENSG00000147403), *RPS3* (ENSG00000149273), and *RPS4X* (ENSG00000198034). *RPL10*, *RPS3*, and *RPS4X* are all ribosomal genes, and *RPL10* gene is a feature gene in B cells. *RPL10* has crucial function in the immune systems of numerous plants and animals (95–97), and thus has potential function in the human immune system. *RPS3* is involved in the positive regulation of activated T cell proliferation (98, 99) and cytokine production and proliferation in T cells (100), and its function correlates with the function of CD4^+^ helper T cells. However, no publication has directly explored the role of *RPS4X* in immune response, but it is presumed to be related to the execution of immune functions by CD4^+^ T cells because of its involvement in protein translation as a ribosomal gene (101). The alteration of gene expression by SARS-CoV-2 infection may partially demonstrate the trend of the alteration of these genes after COVID-19 vaccination. In 2022, an article suggested that the *RPS4X* gene showed downregulated expression after SARS-CoV-2 infection (102). Although no subsequent literature has demonstrated COVID-19 vaccination-induced differential expression of *RPL10*, *RPS3*, and *RPS4X* in CD4^+^ T cells, these genes are still considered potent features.

#### 4.1.3 Qualitative features in CD8+ T cells

The first identified feature was *JUN* (ENSG00000177606), which is involved in the negative host regulation of viral transcription (103) and thus has potential role in immune response. In addition, c-Jun expression is a key component of the JNK/AP-1 pathway, which plays an important role in the regulation of stress response genes with anti-inflammatory and cytoprotection function (104). The immune functions of *JUN* may provide indirect evidence of the differential expression of *JUN* after COVID-19 vaccination. Further, a 2020 paper identified that SARS-CoV-2 infection can cause the differential expression of *JUN* through pathway enrichment analysis and identified the *JUN* as a novel biomarker (105). Therefore, *JUN* may exhibit differential expression after COVID-19 vaccination and may serve as a plausible signature gene.

The next identified feature gene was *MTRNR2L12* (ENSG00000269028), which is a paralog of protein-coding genes and associated with apoptosis (65). Although no paper has discussed the immune function of *MTRNR2L12* in CD8^+^ T cells, the differential expression of *MTRNR2L12* in patients with COVID-19 may provide indirect evidence of COVID-19 vaccination. In 2021, researchers found that *MTRNR2L12* was upregulated in immune cells, such as CD8^+^ T cells, in bronchoalveolar lavage fluid from mild and severe COVID-19 patients (106), suggesting the possibility of altered expression following COVID-19 vaccination. Two recent papers published in 2022 found the differential expression of *MTRNR2L12* in bronchoalveolar lavage fluid samples from patients with severe COVID-19 (107) and classification of the MTRNR2L12 gene as an important gene determining COVID-19 positive status by association classification model (108). Thus, COVID-19 vaccination may cause the differential expression of *MTRNR2L12* and serve as a feature that facilitates the differentiation among the protective capacities of different vaccination strategies.

The next identified gene was *PLCG2* (ENSG00000197943), which is a feature gene that may respond to vaccine protection in B cells. In contrast to what was previously mentioned, *PLCG2* is implicated in the T cell receptor signaling pathway (109). Li et al. also found that *PLCG2* expression is positively correlated with immune cells, such as CD8^+^ T cells (110). The alteration of *PLCG2* expression by natural infection with SARS-CoV-2 has been discussed in the previous section. *PLCG2* expression in CD8^+^ T cells may represent T cell immunological response following COVID-19 vaccination. In summary, *PLCG2* in CD8^+^ T cells can be used as an effective feature.

The next identified feature was *RPS29* (ENSG00000213741), which encodes a ribosomal protein involved in the protein translation (111, 112) and is associated with CD8^+^ T cells that kill infected cells (113). Additionally, differential expression of *RPS29* may partially react to the differential expression of *RPS29* following vaccination in patients with COVID-19. In 2020, Vastrad et al. identified *RPS29* as a biomarker for the diagnosis of SARS-CoV-2 infection through pathway enrichment analysis (114), so *RPS29* expression may be altered after COVID-19 vaccination. In addition, Yang et al. found that ribosome-encoding genes, such as *RPS29*, were specifically downregulated in patients with long duration of toxic shedding. Based on the above discussion, *RPS29* may be differentially expressed in CD8^+^ T cells after COVID-19 vaccination as a feature of the protective power of response vaccine.

The last identified gene was *XIST* (ENSG00000229807), which encodes noncoding RNAs that specifically silences X chromosome (115). *XIST* expression is closely associated with T cells. A study found that the high expression of the *XIST* gene was associated with CD8^+^ T cell and total T cell levels (116). In addition, the high expression of *XIST* stimulates the proliferation and differentiation of naïve CD4^+^ T cells (117). Thus, *XIST* is closely associated with T cell-induced immune response and may be differentially expressed after COVID-19 vaccination. *XIST* can still be a feature in CD8^+^ T cells according to its immunological role even if no study provides clear evidence of differential expression of *XIST* after vaccination.

### 4.2 Analysis of decision rules in lymphocytes for distinguishing among vaccination strategies

As described above, we identified a set of validated features that can help qualitatively distinguish among lymphocyte gene expression samples from various prime-boost vaccination strategies. Some top features have been validated by recent studies. For a more thorough discussion, we selected a few representative rules for each class based on blood single cell data for B, CD4^+^ T, and CD8^+^ T cells, which are listed in **Table 7**. We compared the protective effects of BNT–BNT, BNT–ChAd, and ChAd–ChAd vaccine combinations based on the differential expression of some important genes. Then, the effectiveness of immunity induced by vaccination strategies based on the roles of these genes in B, CD4^+^ T, and CD8^+^ T cells were predicted.

**Table 7:** Representative rules in lymphocytes.

Cell Type	Rules	Parameters	Predicted class
B Cell	Rule 0	[ENSG00000269028 (MTRNR2L12) ≤ 2.68] and [ENSG00000197943 (PLCG2) ≤ 3.90] and [ENSG00000145425 (RPS3A) ≤ 3.49]	BNT-BNT
	Rule 1	[ENSG00000198938 (MT-CO3) >4.84] and [ENSG00000265972 (TXNIP) ≤ 4.73] and [ENSG00000197943 (PLCG2) >0.59] and [ENSG00000145425 (RPS3A) >3.68]	BNT-ChAd
	Rule 2	[ENSG00000198938 (MT-CO3) >4.52] and [ENSG00000269028 (MTRNR2L12) ≤ 4.81] and [ENSG00000145425 (RPS3A) ≤ 3.49]	ChAd-ChAd
CD4^+^ T Cell	Rule 3	[ENSG00000166710 (B2M) ≤ 3.56] and [ENSG00000197728 (RPS26) ≤ 1.49] and [ENSG00000269028 (MTRNR2L12) ≤ 2.51]	BNT-BNT
	Rule 4	[ENSG00000166710 (B2M) >3.56] and [ENSG00000198938 (MT-CO3) >1.83] and [ENSG00000125740 (FOSB) ≤ 3.16]	BNT-ChAd
	Rule 5	[ENSG00000166710 (B2M) ≤ 5.23] and [ENSG00000125740 (FOSB) ≤ 0.53]	ChAd-ChAd
CD8^+^ T Cell	Rule 6	[ENSG00000269028 (MTRNR2L12) ≤ 2.99] and [ENSG00000197943 (PLCG2) ≤ 2.94] and [ENSG00000229807(XIST) ≤ 0.72]	BNT-BNT
	Rule 7	[ENSG00000166710 (B2M) > 3.60] and [ENSG00000115523(GNLY) ≤ 2.06] and [ENSG00000197943 (PLCG2) >1.59] and [ENSG00000125740 (FOSB) ≤ 3.89]	BNT-ChAd
	Rule 8	[ENSG00000197943 (PLCG2) ≤ 5.00] and [ENSG00000198840(MT-ND3) ≤ 5.00]	ChAd-ChAd

#### 4.2.1 Quantitative rules in B cells

*MTRNR2L12* (ENSG00000269028) is downregulated in B cells after two doses of BNT or ChAd vaccination. *MTRNR2L12* is an anti-apoptotic lncRNA (106), and the expression of *MTRNR2L12* is positively correlated with cellular stress (118). Based on the relationship between *MTRNR2L12* expression and cellular stress, we hypothesized that the low expression of *MTRNR2L12* may be associated with decrease in adverse vaccine reactions. A study in 2021 reported a higher incidence of serious adverse events due to ChAd–BNT than that after homologous vaccines (119). Thus, the low expression of *MTRNR2L12* facilitates differentiation among homologous vaccines with low adverse reaction rates.

*PLCG2* (ENSG00000197943) is involved in the B cell receptor signaling pathway (59) and associated with antibody production and B cell memory (60). Therefore, the expression of *PLCG2* is altered after COVID-19 vaccine administration. In addition, the expression level of *PLCG2* may suggest the effectiveness of humoral immunity induced by different vaccine combinations. In 2022, a study found that homologous BNT–BNT-induced lower anti-S IgM and IgG concentrations to a higher degree than heterologous BNT–ChA (120). Similarly, Pozzetto et al. (15) found that heterologous ChAd–BNT vaccination strategy produced more effective neutralizing antibodies than vaccination with homologous BNT-BNT. Thus, *PLCG2* is useful in identifying ChAd–BNT vaccine recipients.

*RPS3A* (ENSG00000145425) is overexpressed after BNT–ChAd vaccination. The small ribosomal subunit (40S) contains RPS3A, which is primarily found in the cytoplasm and nucleus (121). *RPS3A* plays a critical role in regulating translation initiation and protein synthesis (122, 123), so the expression of *RPS3A* may be related to antibody secretion. In 2022, a study found that BNT–ChAd vaccination produced more anti-S IgG than BNT–BNT vaccination in people without previous SARS-CoV-2 infection (124). In 2021, another study found that BNT–ChAd induced higher titers of anti-S protein IgG and IgA subclasses than a homologous vaccination strategy (16), confirming that *RPS3A* is a valid parameter for predicting people receiving heterologous BNT–ChAd vaccines.

High expression of *MT-CO3* (ENSG00000198938) is associated with vaccination with heterologous BNT–ChAd vaccines. *MT-CO3*, a mitochondrial gene (65), enables B cells to obtain sufficient energy to perform their functions. When B cells are activated by an antigen and differentiate into plasma cells to secrete antibodies, a high level of oxidative phosphorylation is required (125). Therefore, the upregulation of *MT-CO3* is reasonable given that COVID-19 vaccination induces B-cell-mediated humoral immunity (126, 127). In 2021, a study found that initial booster vaccination with heterologous BNT–ChAd induced the production of high concentrations of anti-S IgG (128). In addition, BNT–ChAd vaccinees produce more anti-RBD IgG than ChAd–ChAd vaccines (129). The effectiveness of *MT-CO3* upregulation was demonstrated by the fact that heterologous vaccines induce the production of a large number of antibodies and therefore require a large energy supply.

*TXNIP* (ENSG00000265972) is downregulated after BNT–ChAd vaccination, helping to identify homologous vaccinated individuals. The product encoded by *TXNIP* can inhibit the activity of Trx1, thereby suppressing rapid cellular proliferation (130). Thus, *TXNIP* can inhibit the proliferation of B cells during an immune response. Immune response to vaccination causes B cell proliferation, indicating the downregulation of *TXNIP* in B cells. In view of the strong humoral immune response induced by BNT–ChAd prime-boost vaccination reported in 2021 (15), low *TXNIP* expression is associated with heterologous vaccination.

#### 4.2.2 Quantitative rules in CD4^+^ T cells

*B2M* (ENSG00000166710) is a marker of immune activation and involved in the positive regulation of T cell activation (88). Therefore, *B2M* expression in CD4^+^ T cells may be related to CD4^+^ T cell activation and executive functions. Schmidt et al. found that the homologous vaccination strategy resulted in lower IFN-γ level than the heterologous vaccination strategy (10, 129), demonstrating a weaker CD4^+^ T cell immune response in BNT–BNT vaccinees. Thus, the overexpression of *B2M* in CD4^+^ T cells can facilitate distinction of BNT–ChAd prime-boost vaccination from other types of vaccination.

*RPS26* (ENSG00000197728) has low expression to predict BNT–BNT vaccines. *RPS26* is a ribosomal protein-encoding gene and plays a key role in regulating T cell survival (131). The expression of *RPS26* is related to T cell-mediated cellular immunity. In addition, in 2022, a study found differential expression of *RPS26* after COVID-19 mRNA vaccination (132), demonstrating the validity of *RPS26* as a parameter. In 2021, another study demonstrated that the BNT–BNT vaccination strategy induced less spike-specific IFN-γ than the BNT–ChAd vaccination strategy did (133). Therefore, the downregulation of *RPS26* is associated with the identification of BNT–BNT vaccine recipients.

In B cells, the low expression of *MTRNR2L12* (ENSG00000269028) is associated with a low incidence of adverse reactions to two doses of BNT vaccine and with the identification of a BNT+BNT vaccination strategy. As previously explained, a low incidence of adverse reactions to two doses of the BNT vaccine is related to the low expression of *MTRNR2L12*. Therefore, *MTRNR2L12* can also be used as a valid parameter in CD4^+^ T cells.

The expression of *MT-CO3* (ENSG00000198938) in CD4^+^ T cells facilitates the identification of a group that has received heterologous BNT–ChAd vaccination. We have already discussed MT-CO3 as a mitochondrial gene in B cells engaged in ATP synthesis. A study in 2021 suggested that the BNT–ChAd immunization method produced great protection (134), suggesting that *MT-CO3* is a useful parameter in CD4^+^ T cells.

*FOSB* (ENSG00000125740) is downregulated after heterologous BNT–ChAd vaccination. *FOSB* is an AP-1 family transcription factor that participates in the regulation of T cell proliferation, differentiation, and immune response (135). In 2022, a study found that the *FOSB*-encoded AP-1 transcription factor was downregulated after BNT vaccination (136), suggesting the extent at which *FOSB* downregulation may facilitate distinction among different vaccine strategies. Although no publications have proven the validity of *FOSB*, *FOSB* can still be identified as a parameter in this rule.

#### 4.2.3 Quantitative rules in CD8^+^ T cells

*MTRNR2L12* (ENSG00000269028) contributes to the low incidence of adverse reactions after BNT–BNT vaccination (119, 137) and is thus a valid parameter in CD8^+^ T cells and facilitates the differentiation of a BNT–BNT vaccination population from another population.

*PLCG2* (ENSG00000197943) expression is positively correlated with CD8^+^ T cells (110), and the protective capacity of homologous vaccination is lower than that of BNT–ChAd vaccination (128), *PLCG2* can be used as a valid parameter in CD8^+^ T cells for identifying individuals with BNT–ChAd vaccines.

The low expression of *XIST* (ENSG00000229807) may be the result of the reduced level of response of CD8^+^ T cells after BNT–BNT vaccination because a high expression of *XIST* increases the amounts of CD8^+^ T cells (117). In 2021, a study found that BNT–BNT vaccination produced less IFN-γ than heterologous vaccination (133), also demonstrating that the expression of *XIST* in CD8^+^ T cells can facilitate the identification of people who have received two doses of the BNT vaccine.

The upregulated expression of *B2M* (ENSG00000166710) in CD8^+^ T cells facilitates the identification of BNT–ChAd vaccination strategies. The *B2M* gene is involved in T cell-mediated cytotoxicity (90) and T cell activation (65), and so *B2M* plays an important role in the immune function of CD8^+^ T cells. Given that the heterologous BNT–ChAd vaccination strategy induces stronger cellular immunity than the homologous vaccination strategy does (15, 129), *B2M* can be regarded as a parameter.

*GNLY* (ENSG00000115523) expression in CD8^+^ T cells is useful in predicting BNT–ChAd vaccine recipients. *GNLY* is a cytotoxicity-associated gene involved in CD8^+^ T cell-mediated protective immunity (138, 139). However, the low expression of *GNLY* in this rule may be due to the fact that *GNLY* is released by CD8^+^ T cells to kill cells infected by SARS-CoV-2. The BNT–ChAd vaccination strategy induces stronger cellular immunity (128), and thus *GNLY* facilitates the identification of individuals with the BNT–ChAd vaccine.

The expression of *FOSB* (ENSG00000125740) in CD8^+^ T cells helps in identifying people who received heterologous BNT–ChAd vaccination. In 2021, a study found high *FOSB* expression in senescent T cells (140), presumably with few senescent CD8^+^ T cells due to the induction of CD8^+^ T cell proliferation by vaccination with BNT–ChAd vaccine. In addition, the BNT–ChAd vaccination strategy induces stronger cellular immunity than the homologous vaccination strategy does (16), and thus *FOSB* can serve as an effective parameter in CD8^+^ T cells.

*MT-ND3* (ENSG00000198840) is a mitochondrial gene associated with the energy supply of CD8^+^ T cells performing immune functions. The downregulation of *MT-ND3* CD8^+^ T cells allows the identification of homologous ChAd–ChAd vaccination strategies, as BNT–ChAd heterologous vaccines induce strong immune responses (134). Therefore, *MT-ND3* can be regarded as a valid parameter in CD8^+^ T cells.

## 5 Conclusion

In the present study, a set of potential genes that reveal differential expression in B, CD4^+^ T, and CD8^+^ T cells induced by COVID-19 vaccination were identified. The genes may facilitate distinction among the immunological effects of BNT–BNT, ChAd–ChAd, and ChAd–BNT vaccinations. The differential expression of the features we identified in subjects vaccinated with different COVID-19 vaccine types may provide evidence of the protective capacities of different vaccination strategies and help advance effective vaccination methods, providing protection against SARS-CoV-2 infection. According to newly released publication, some features and quantitative rules were associated with COVID-19 vaccination and SARS-CoV-2 infection. Meanwhile, some efficient classifiers with the screened features were set up, indicating that selected features can effectively distinguish between heterologous and homologous vaccines. The high efficacy of the heterologous ChAdOx1–BNT162b2 vaccine can be partly explained by this study, which offers a theoretical foundation for vaccine modification.

## Data availability statement

Publicly available datasets were analyzed in this study. This data can be found here: https://www.ncbi.nlm.nih.gov/geo/query/acc.cgi?acc=GSE201534.

## Author contributions

TH and Y-DC designed the study. JL, WG, and KF performed the experiments. FH and QM analyzed the results. JL, FH, and QM wrote the manuscript. All authors contributed to the research and reviewed the manuscript. All authors contributed to the article and approved the submitted version.

## Supplementary material

Supplementary Table 1 List of features in three immune cell types sorted by LASSO, LightGBM, mRMR, MCFS, and PFI methods.

Supplementary Table 2 IFS results of DT and RF on lists yielded by different feature ranking algorithms in three immune cell types.

Supplementary Table 3 Venn results for essential features, used in feasible classifiers (if available) or optimal classifiers, identified by five feature ranking algorithms in three immune cell types.

Supplementary Table 4 Classification rules based on the optimal DT classifiers created in three immune cell types. Each rule is composed of gene symbol and its expression level and describes how the expression level determines the class labels of samples.
